# *Enterobacter*-inoculation altered the C, N contents and regulated biomass allocation in *Reaumuria soongorica* to promote plant growth and improve salt stress tolerance

**DOI:** 10.3389/fpls.2024.1502659

**Published:** 2025-01-03

**Authors:** Xin-Guang Bao, Pei-Fang Chong, Cai He, Xue-Mei Lu, Xue-Ying Wang, Feng Zhang, Bing-Bing Tan, Jia-Li Yang, Li-Li Gao

**Affiliations:** ^1^ Key Laboratory of Grassland Ecosystem, Gansu Agricultural University, Ministry of Education, Lanzhou, China; ^2^ College of Forest of Gansu Agriculture University, Lanzhou, China; ^3^ Institute for Desertification Control and Prevention, Wuwei Academy of Forestry, Wuwei, China; ^4^ Minqin County Liangucheng Psammophytes Nature Reserve Management Station, Wuwei, China

**Keywords:** *Reaumuria soongorica*, *Enterobacter*, salt stress, biomass allocation, stoichiometry

## Abstract

Soil salinization poses a significant ecological and environmental challenge both in China and across the globe. Plant growth-promoting rhizobacteria (PGPR) enhance plants’ resilience against biotic and abiotic stresses, thereby playing a vital role in soil improvement and vegetation restoration efforts. PGPR assist plants in thriving under salt stress by modifying plant physiology, enhancing nutrient absorption, and synthesizing plant hormones. However, the mechanisms through which PGPR regulate the contents of carbon (C) and nitrogen (N), and biomass allocation of desert plant in response to salt stress is still unclear. This study explores the impact of PGPR on biomass allocation, C, and N contents of *R. soongorica* seedlings through a pot experiment. Strains P6, N20, and N21, identified as *Enterobacter*, were isolated from the rhizosphere of *R. soongorica*, and they exhibited various beneficial traits such as indole-3-acetic acid (IAA) production, phosphate solubilization, nitrogen fixation, and tolerance to up to 8% NaCl stress. We found that under NaCl stress, *R. soongorica* seedlings exhibit significant reductions in plant height, basal diameter, and root surface area (*P*<0.05). However, inoculation with strains P6, N20, and N21 reverses these trends. Compared to NaCl treatment alone, co-treatment with these strains significantly increases the biomass of roots, stems, and leaves, particularly root biomass, which increases by 99.88%, 85.55%, and 141.76%, respectively (*P*<0.05). Moreover, N contents decrease significantly in the roots, stems and leaves, C contents increase significantly in the roots and leaves compared to NaCl treatment (*P<*0.05). Specifically, N contents in roots decrease by 14.50%, 12.47%, and 8.60%, while C contents in leaves increase by 4.96%, 4.45%, and 4.94%, respectively (*P*<0.05). Additionally, stem and leaf biomasses exhibit a significant positive correlation with C contents and a significant negative correlation with N contents in these tissues. In conclusion, inoculation of *Enterobacter* strains enhanced the biomass of *R. soongorica* seedlings, regulated the biomass distribution, and modifies C and N contents to promote plant growth and improve salt stress tolerance. This study provides a novel adaptive strategy for the integrated use of PGPR and halophytes in saline-alkali soil improvement and vegetation restoration efforts.

## 1 Introduction

Land desertification is an ecological and environmental problem in China and globally. Water scarcity, salinization, and lack of nutrients in desert soils are the main constraints on the recovery and reconstruction of vegetation in desert areas, which seriously threaten the growth of desert plants (Hidri et al., 2019; Zhang et al., 2019). Under adversity, plants respond to environmental stress through various adaptive strategies and survival mechanisms, such as changes in their own morphology, physiological and biochemical responses, metabolic pathways, and hormonal regulation (Chen et al., 2019; Deinlein et al., 2014; Sun et al., 2019). In addition, plants can respond to environmental changes by adjusting the allocation of biomass to different organs. According to several studies, when plants encounter water and nutrient stress, more biomass will be allocated to the root (Chen et al., 2019; Grechi et al., 2007; Sun et al., 2019). However, under low light and CO_2_ conditions, plants allocate a higher proportion of biomass to their aboveground parts (Guo et al., 2016), which is consistent with the assumptions made by the theory of optimal allocation (Asefa et al., 2022). However, salt stress reduces the rate of photosynthesis, and growth rate, inhibits biomass accumulation, and alters biomass partitioning patterns in plants (Ilangumaran and Smith, 2017). As prevalent stress in desert areas, salt stress limits plant growth and yield (Hidri et al., 2022; Komaresoflaa et al., 2019). Vegetation restoration and preservation stand out as effective measures to counter desertification. Saline plants, as the dominant species in desert areas, play an important role in land reclamation and vegetation restoration (Najafi Zilaie et al., 2022).

*R. soongorica* is a salt-secreting xerophyte shrub of *Tamaricaceae*, which is one of the typical dominant shrub species in arid and semi-arid desert areas in northwest China (Bai et al., 2009; Ma et al., 2005; Shi et al., 2013). It is also a key species for windbreaks, sand fixation, and maintaining the stability of desert ecosystems (Chong et al., 2019). In the process of maintaining the ecological balance in desert areas, *R. soongorica* has evolved special physiological characteristics to withstand environmental factors such as drought and salinity (Shi et al., 2013). It has become an important ecological “barrier” for the protection of decertified land. Therefore, many scholars have studied its morphology and structure (Chong et al., 2019; Liu et al., 2018), vegetation restoration and drought adaptation strategies (Shan et al., 2015), salt-tolerance mechanisms (Liu et al., 2023; Yan et al., 2022a), and distribution pattern and artificial seedling. It provides a theoretical basis for the wide application of *R. soongorica* in desertification control. However, the slow growth of *R. soongorica* at the seedling stage, its weak stress resistance, and its low survival rate have hindered widespread adoption in artificial vegetation projects in desert regions. This limitation significantly impacts ecological restoration and sustainable progress in decertified zones (He, 2019). Therefore, urgent measures are needed to bolster the resilience of *R. soongorica* seedlings in saline and alkaline environments, enhance their survival chances, and foster healthy growth.

Currently, the utilization of plant growth-promoting rhizobacteria (PGPR) in agriculture and forestry to promote plant growth, yield, and stress resistance is widely considered an environmentally friendly, sustainable, and cost-effective approach (Dong et al., 2024). PGPR is a soil bacterium that settles in plant roots and promotes plant growth. It can mediate the increase of soil nitrogen, phosphorus, and potassium nutrient availability and produce plant hormones to directly promote plant growth (Amri et al., 2023; Li et al., 2022b; Mukherjee and Sen, 2015). It can also indirectly support plant growth by alleviating the harmful effects of biotic and abiotic stresses (El-Beltagi et al., 2022; Peng et al., 2023). There is also evidence that PGPR resists biotic and abiotic stresses by regulating plant biomass allocation. The study found that the root and tiller of wheat were allocated a larger proportion of biomass by inoculating microbial inoculants, which offset the negative impact of Hessian flies on wheat (Prischmann-Voldseth et al., 2020). Inoculation of PGPR and arbuscular mycorrhizal fungi(AMF) alleviates the negative effects of drought on the biomass and morphological and physiological characteristics of sweet peaches (Azizi et al., 2022). Inoculation of rhizosphere microorganisms can change the content of C and N in plants, help to adapt to environmental changes, improve plant nutrition, and resist abiotic stress damage (Mariotte et al., 2017). Modulation of C:N:P stoichiometry is involved in the effectiveness of a PGPR and AMF in increasing salt stress tolerance of *Sulla carnosa* (Hidri et al., 2019). Thus, PGPR plays a key role in plant growth and development as well as in addressing various environmental stresses, but it mainly focuses on crops and economic forests, whereas minimal research has been conducted on desert shrubs. There was no report on the effects of PGPR on the biomass allocation and C, N content of roots, stems, and leaves from *R. soongorica* seedlings. Therefore, in this study, three *Enterobacter* strains were selected from the rhizosphere of the desert plant *R. soongorica* in northwest China, and their effects on the biomass allocation of the host and the contents of C and N in different organs were verified through pot experiments. It is of great significance to develop potential bio inoculants that are beneficial to the growth of desert plants and the bioremediation of saline-alkali land.

## 2 Materials and methods

### 2.1 Test strains and inoculum preparation

P6, N20, and N21 were selected as the experimental strains for this study based on their growth-promoting capabilities, including IAA production, nitrogen fixation, and phosphorus solubilization. These strains were isolated from the desert shrub *R. soongorica* rhizosphere soil of Qingtu Lake in the Shiyang River Basin, Northwest China by Li et al. (2020). They were stored in Luria-Bertani(1% tryptone, 0.5 yeast extract, 1% NaCl, LB) broth containing 25% glycerol at −80°C. The bacterial inoculums were prepared by growing every strain in LB at 28°C for 48 h. Each bacterial culture was harvested and diluted with sterile physiological saline (0.9% NaCl solution) to a final OD of 1 at 600 nm (~ 10^9^ CFU mL^−1^).

### 2.2 *R. soongorica* seedling cultivation and growth conditions

For this experiment, utilized *R. soongorica* seeds were collected from Qingtu Lake located in Minqin County in 2021 and preserved in a dry refrigerator maintained at 0-4°C for an extended period. Prior to planting, the seeds underwent sterilization using a 1% sodium hypochlorite solution for half an hour, followed by five rinses with sterile water. In April 2023, these sterile seeds were sown in trays. The seedlings were transplanted into plastic flower pots with a diameter of 24.8 cm and a height of 28 cm, with one plant per pot, when they reached a height of 5 cm. The plastic flower pots contained 5 kg of a substrate mixture of clay, sand, and humus (1:2:1), soil organic matter(SOM): 28.84 g·kg^-1^, total carbon: 37.81 g·kg^-1^, total nitrogen: 0.98 g·kg^-1^, (TP): 0.30 g·kg^-1^, available phosphorus (AP): 14.71: mg·kg^-1^, total potassium (TK): 3.57 g·kg^-1^, available potassium (AK): 105.75 mg·kg^-1^, and electrical conductivity (EC): 0.32 mS·cm^−1^. *R. soongorica* seedlings grew under natural conditions at the experimental site of Gansu Agricultural University, located in Anning District, Lanzhou City, Gansu Province (36°5′N, 103°42′E), which is in a mid-temperate climate zone with distinct inland climate characteristics, clear seasonal changes, ample sunlight, and dry weather.

### 2.3 Physiological and biochemical identification and NaCl tolerance test of three strains

Three strains were activated on the LB solid medium and incubated at 30°C for 48 hours. Gram staining was performed according to the instructions of the Biosharp Gram Staining Kit. The physiological and biochemical properties of the strains were determined by referring to Bergey’s Bacteria Identification Manual and Manual of Identification of Common Bacterial Systems (Buchanan and Gibbons, 1984; Dong and Cai, 2001). Three strains were inoculated on LB solid medium containing 4%, 6%, 8%, 10%, and 12% NaCl to test the NaCl tolerance of strains.

### 2.4 Plant growth-promoting function test of strains

The abilities of the strains to produce IAA were evaluated using a colorimetric assay (Bao et al., 2024; Kumar and Audipudi, 2015). According to Nautiyal (1999) and Amri et al. (2023), the abilities of the strains to solubilize organic and inorganic phosphorus were tested and were expressed by the size of the phosphorus dissolving circle around the colony. The strains were inoculated onto Ashby medium and considered to have nitrogen-fixing ability if they could still grow on Ashby medium after three successive transfers (Bao et al., 2024). All the above tests were repeated three times.

### 2.5 Molecular identification of strains

The strains were activated with LB solid medium, and incubated at 30°C for 24 hours. Use the bacterial DNA extraction kit to extract Bacterial DNA. The 16S rDNA sequences of the strains were amplified with primers 27F (5’-AGAGTTTGATCMTGCTCAG-3’) and 1492R (5’-TACGGYTACCTTGTTACGATT-3’). Then, PCR products were sent to Guangdong Magigene Biotechnology Co., Ltd. In Guangzhou, China for Sanger sequencing. Finally, the 16S rRNA gene sequences were analyzed using the EzTaxon (https://www.ezbiocloud.net/apps) online database. A phylogenetic tree was constructed using the Maximum Likelihood Estimate method in MEGA 11.0 software, calculating evolutionary distances with the General Time Reversible (GTR) model, with bootstrap testing repeated 500 times to determine the taxonomic status of the isolated strains (Bao et al., 2024).

### 2.6 Experimental design and treatments

The test set up control (CK, without NaCl and strains), single salt treatment (S, only NaCl added), separate treatment of P6, N20, and N21 bacteria (P6, N20, N21), and co-treatment of salt and bacteria (P6+S, N20+S, N21+S). Each treatment had 6 replicates, 3 replicates for biomass determination, and 3 replicates for C and N content determination. According to reports, a low concentration (100 mM) of NaCl can stimulate the growth of *R. soongorica*, whereas higher concentrations (400 and 500 mM) significantly hinder its development (Yan et al., 2022b). Furthermore, exposure to 500 mM NaCl stress can result in the death of individual *R. soongorica* seedlings (Yan et al., 2022b). Consequently, a concentration of 400 mM NaCl was chosen for treating the seedlings in this study. To avoid osmotic shock, 500 mL of NaCl solution was poured into each basin per day for 3 consecutive days, with a total amount of 1500 mL, and the control were treated with the same amount of water. Trays were placed at the base of the pots to collect any excess water. After watering, the oozing water was poured into the pots again to prevent salt loss. The roots of the plants were inoculated with 100 mL of inoculums by pouring, and the control was inoculated with an equal amount of sterile saline.

### 2.7 Determination of growth indexes of *R. soongorica* seedlings

Samples were taken after the seedlings were treated for 56 days. Before sampling, Photographs were taken with a digital camera, and height and basal diameter were measured for all *R. soongorica* seedlings; Then samples, and collect the plants and the aboveground and underground parts respectively, were back to the laboratory with a low-temperature sampling box, clean them with pure water, and then absorb the surface water with filter paper, and weigh the fresh weight respectively. Separate the roots, stems, and leaves, blanch them at 105 °C for 30 min, and dry them at 80 °C to constant weight. Then, use a digital balance with a progress of 0.0001g to measure the biomass of roots, stems, and leaves. The root length and surface area were determined using the LA-S root analysis system (WSEEN, China).

### 2.8 Determination of the contents of C, N in roots, stems and leaves from the *R. soongorica*

Crush the dried samples and pass them through a 100-mesh sieve for standby. The contents of N and C in roots stems, and leaves were measured by the Carbon and Nitrogen analyzer (Primacs^SNC-100^, Skalar Co., the Netherland).

### 2.9 Statistical analysis

All experiments were designed with three replicates and analyzed using IBM SPSS Statistics 26.0 software for one-way analysis of variance (ANOVA). Duncan’s minimum significant difference method was used to assess the significance of differences among treatments, and the significance level was set at 0.05. The graphs were created using Origin 2021 and Adobe Illustrator 2022 software. Pearson correlation coefficient (r) and linear regression were used to analyze the correlation between the biomass allocation and the C, N content of roots stems, and leaves.

## 3 Results

### 3.1 Physiological and biochemical properties and plant growth-promoting function of strains

The main physiological and biochemical characteristics and growth-promoting functions of strains P6, N20, and N21 are shown in **Table 1**. Strains P6, N20, and N21 are all gram-negative bacteria. It is positive under the Voges-Proskaue test, Indole test, and, Catalase test of these strains, but negative for Methyl red test, Starch hydrolysis test, and Gelatin hydrolysis test. In addition, their glucose fermentation tests are F type. Strains P6, N20, and N21 can grow in the highest NaCl concentration at 8%. Strains P6, N20, and N21 have nitrogen fixation ability, produce growth elements, and dissolve organic phosphorus and inorganic phosphorus.

**Table 1:** Physiological and biochemical properties and the growth-promoting function of the strains.

Strain number	E. coli	P6	N20	N21
Physiological and biochemical properties				
Maximal tolerable dose of NaCl (%)	8%	8%	8%	8%
Glucose fermentation test	Type F	Type F	Type F	Type F
Gram staining	–	–	–	–
Methyl red test	+	–	–	–
Voges-Proskauer test	–	+	+	–
Indole test	+	+	+	+
Starch hydrolysis test	–	–	–	–
Catalase test	+	+	+	+
Gelatin hydrolysis test	–	–	–	–
Plant growth-promoting function				
IAA (µg mL^-1^)	20.66 ± 0.88 d	82.56 ± 0.64 b	87.04 ± 1.14 a	79.74 ± 0.73 c
Nitrogen fixation	***	***	**	**
Inorganic phosphorus solubilization (D/d)	nil	1.79 ± 0.15 a	1.49 ± 0.05 c	1.67 ± 0.09 bc
Organic phosphorus solubilization (D/d)	nil	1.72 ± 0.08 c	2.14 ± 0.18 a	1.81 ± 0.16 bc

D/d, diameter halo/diameter colony; IAA, indole acetic acid; +, presence of activity; -, absence of activity; “**” represents “growth” and “***” represents “good growth”. Mean values labeled with the same superscript letter within the same line were not significantly different at *P<* 0.05 following Duncan’s test; E. coli, due to false positives in the identification of the growth promoting function of PGPR strains, we chose E. coli as positive control.

### 3.2 Identification results of strains

**Figure 1:**
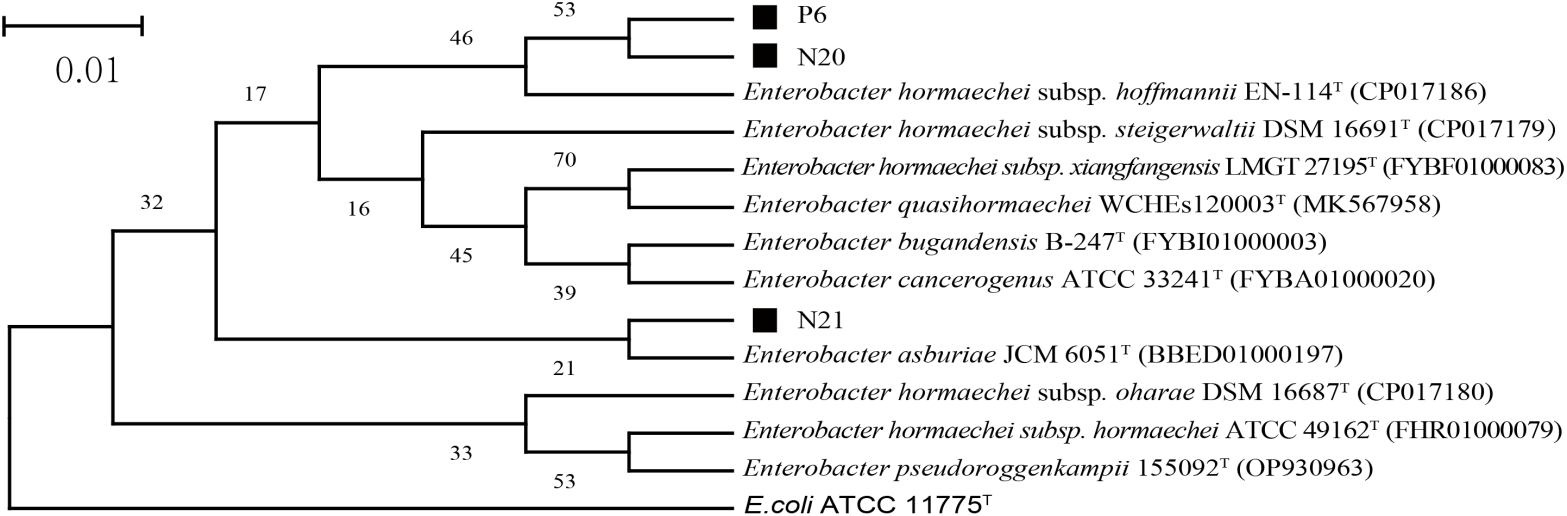
The phylogenetic tree of strains P6, N20, and N21 is based on 16S rDNA sequencing results. Here, *E. coli* ATCC 11775^T^ is an outgroup; Superscript T are type strains; The scale length is 1% base difference.

The 16S rDNA sequencing results of strains P6, N20, and N21 were compared and analyzed. The phylogenetic tree based on the 16S rRNA gene sequences, as shown in **Figure 1**, indicated that the dominant strains P2, N20, and N21 belong to *Enterobacter*, which was consistent with morphological identification results. In addition, the gene fragment of strain P6 was 1374 bp in length, which was the same branch as *Enterobacter hormaechei* subsp. *Hoffmannii* EN-114^T^ and had a similarity of 99.78%. The gene sequence length of strain N20 was 1374 bp, belonging to the same branch as *E. hormaechei* subsp. *Hoffmannii* EN-114^T^, with a similarity of 99.56%. The gene fragment of strain N21 was 1371 bp, belonging to the same branch as *Enterobacter asburiae* JCM 6051^T^, with a similarity of 99.20%. So, combining morphological and molecular biological characteristics, P6, N20, and N21 could be preliminarily determined as *E. hormaechei, E. hormaechei, E. asburiae*, respectively. The 16S rDNA sequences of P6, N20, and N21 strains have been deposited in the NCBI database with GenBank accession numbers PP998345, PP998346, and PP998347, respectively.

### 3.3 *Enterobacter* improved growth and enhanced salt tolerance of *R. soongorica* seedlings

**Figure 2:**
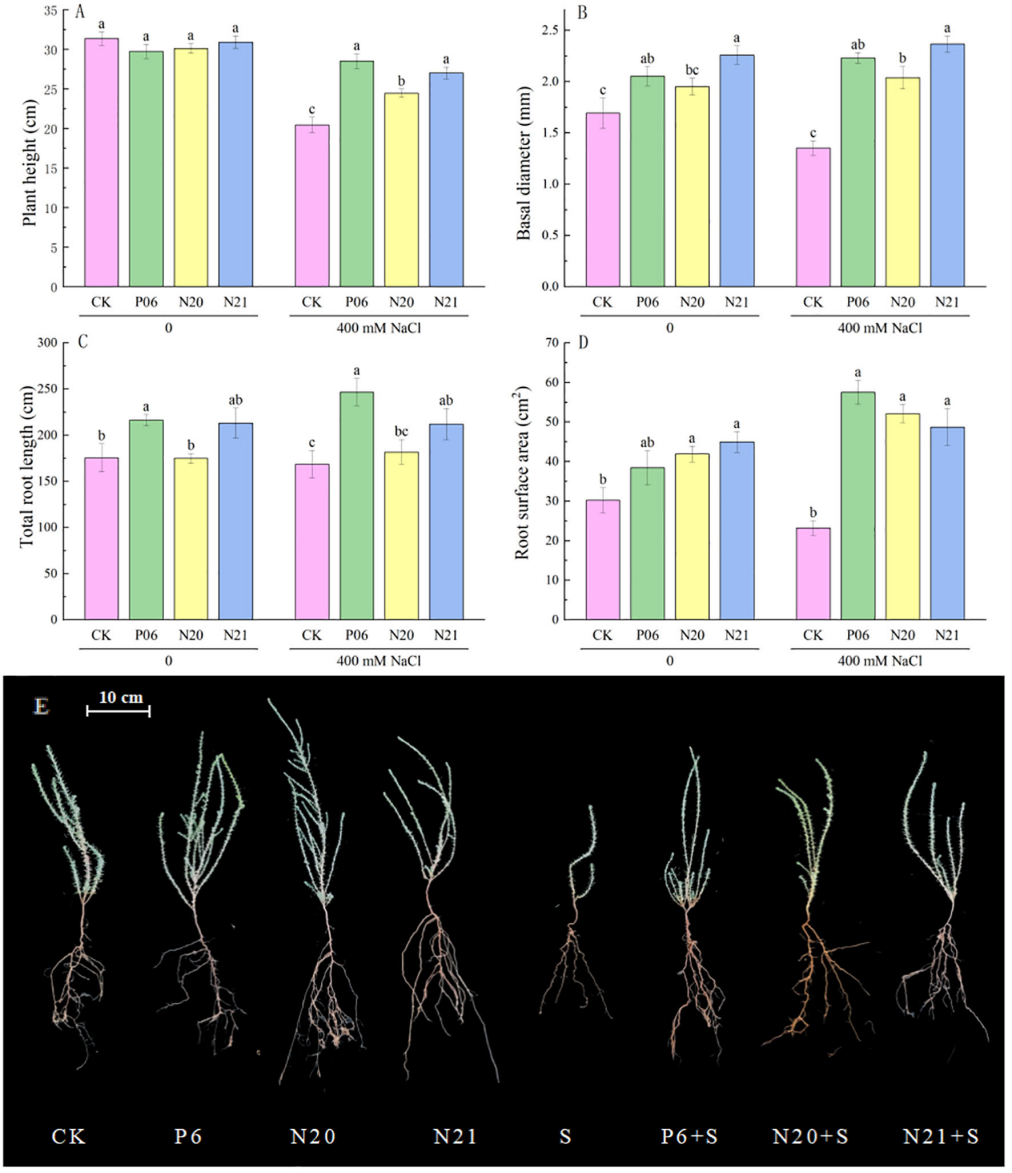
Effects of three *Enterobacter* strains on the growth of *R. soongorica* seedlings under NaCl stress. Mean values labeled with the same superscript letter within the same line were not significantly different at *P< 0.05* following Duncan’s test. **(A)** Plant height, **(B)** Basal diameter, **(C)** Total Root Length, **(D)** Root surface area, and **(E)** Representative pictures of *R. soongorica* seedlings.

Inoculation of three strains of *Enterobacter* has a significant impact on the growth and development of *R. soongorica* and salt tolerance (**Figure 2**). When inoculated with strains P6 and N21, the basal diameters of *R. soongorica* seedlings significantly increased by 21.26% and 33.46% (*P<*0.05) compared to the control (CK), the total root length increased by 23.26% and 21.52%. When inoculated with strains N20 and N21, the root surface area of *R. soongorica* seedlings significantly increased by 38.74% and 48.71% (*P<*0.05) compared with the control (CK). Under NaCl stress, the plant height, basal diameter, and root surface area of *R. soongorica* seedlings were significantly reduced, but when inoculated with P6, N20, and N21 strains, the plant height of *R. soongorica* seedlings significantly increased by 39.41%, 19.54% and 32.08% respectively (*P<*0.05) compared with NaCl treatment (S); the basal diameter significantly increased by 65.02%, 50.74% and 74.88% respectively (*P<*0.05), and the root surface area significantly increased by 147.97%, 124.49% and 109.98% respectively (*P<*0.05) compared with NaCl treatment (S). Moreover, inoculation with strains P6 and N21, the total root length of *R. soongorica* seedlings significantly increased by 46.34% and 25.81% (*P<*0.05) compared with NaCl treatment (S). Thus, inoculation with strains P6, N20, and N21 increased the basal diameters, total root length, and root surface area of *R. soongorica* seedlings, and the effects were more obvious under NaCl stress.

### 3.4 Effect of *Enterobacter* on the biomass of different organs of *R. soongorica* seedlings

**Figure 3:**
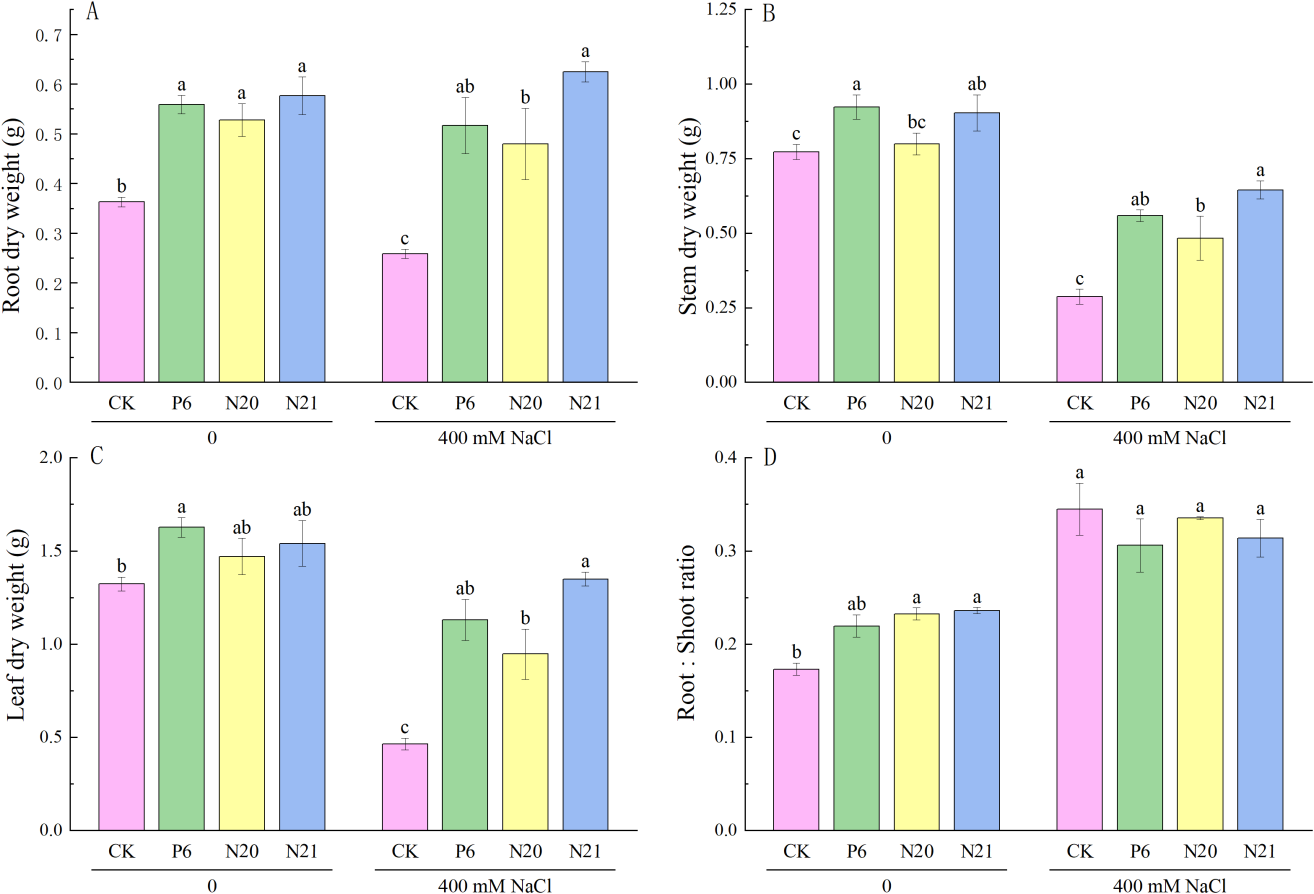
Effects of three *Enterobacter* strains on the biomass of *R. soongorica* seedlings under NaCl stress. **(A)** Root dry weight, **(B)** Stem dry weight, **(C)** Leaf dry weight, **(D)** Root:Shoot ratio. Mean values labeled with the same superscript letter within the same line were not significantly different at *P*< 0.05 following Duncan's test.

Inoculated with *Enterobacter* increased the dry weight of roots, stems, and leaves of *R. soongorica* seedlings, both with and without NaCl stress (see **Figure 3**). Compared with the control (CK), the root dry weight of *R. soongorica* seedlings inoculated with strains P6, N20, and N21 increased by 54.03%, 45.40%, and 58.97% respectively (*P<*0.05); inoculation with strains P6 and N21, the stem dry weight significantly increased by 19.38% and 16.90% (*P<*0.05); inoculation with strains N20 and N21, the root dry weight significantly increased by 34.24% and 36.34% (*P<*0.05). Under NaCl stress, the dry weight of roots, stems and leaves of *R. soongorica* seedlings significantly decreased by 28.73%, 62.83%, and 64.91% (*P<*0.05) compared with the control (CK), and the root-shoot ratio significantly increased by 99.20% (*P<*0.05). Inoculated with strains P6, N20, and N21, the roots dry weight of *R. soongorica* seedlings under NaCl stress significantly increased by 99.88%, 85.55%, 141.76% (*P<*0.05), the stems dry weight significantly increased by 94.60%, 68.33% and 124.59% (*P<*0.05), and leaves dry weight significantly increased by 143.58%, 103.88% and 190.56% (*P<*0.05) compared with NaCl treatment (S). However, there was no significant difference in the root-shoot ratio of *R. soongorica* seedlings. The results showed that strains P6, N20, and N21 could effectively improve the growth and enhance salt tolerance of *R. soongorica* seedlings by regulating biomasses of roots, stems, and leaves. Thus, *Enterobacter* P6, N20, and N21 have the potential as a microbial inoculant that can protect plants under salt-stress conditions.

### 3.5 Effects of *Enterobacter* on C and N contents in different organs of *R. soongorica* seedlings

**Figure 4:**
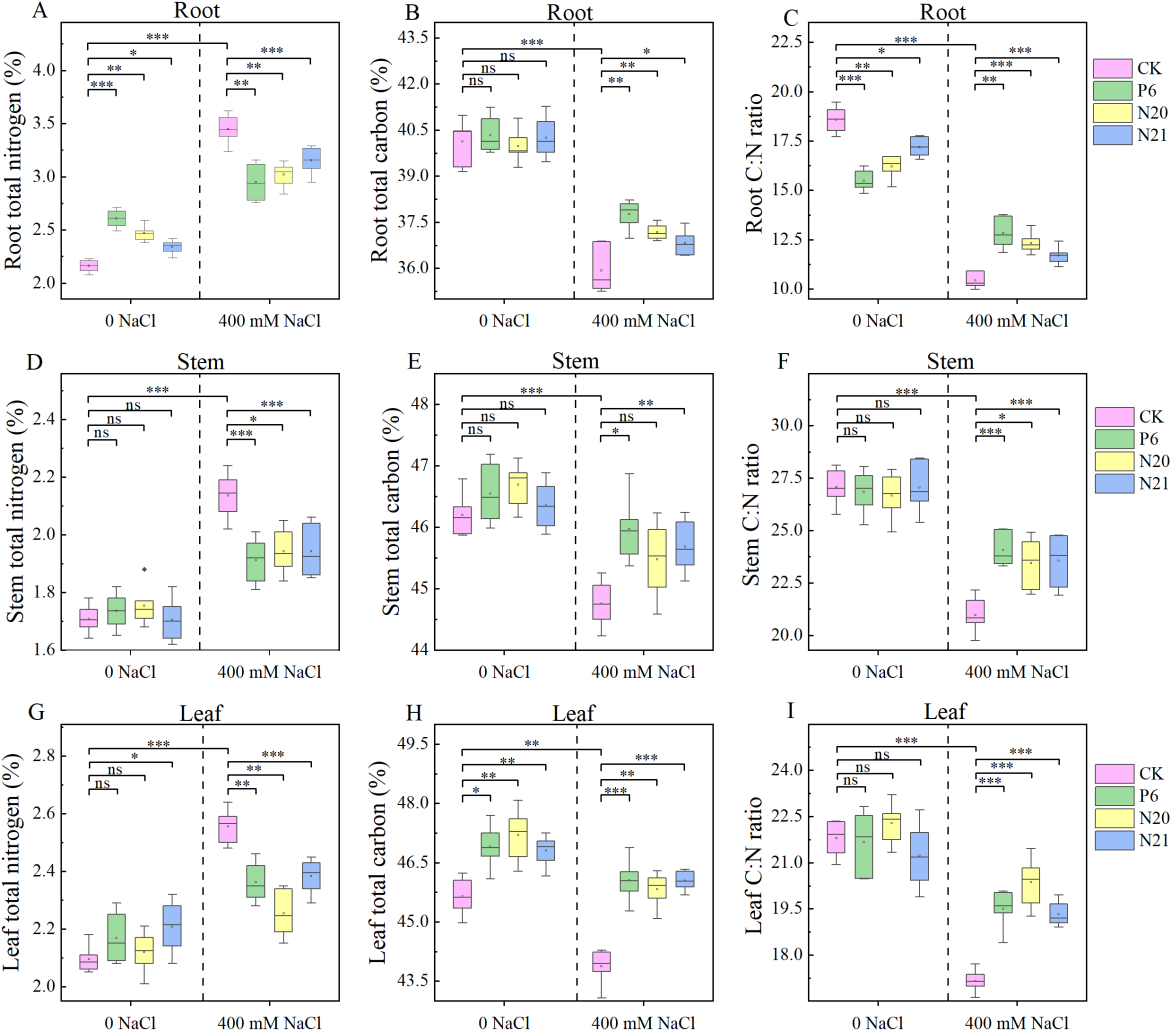
Effects of three *Enterobacter* strains on the C, N contents, C: N ratio of *R. soongorica* seedlings under NaCl stress. **(A)** Root total nitrogen, **(B)** Root total carbon, **(C)** Root C:N ratio, **(D)** Stem total nitrogen, **(E)** Stem total carbon, **(F)** Stem C:N ratio, **(G)** Leaf total nitrogen, **(H)** Leaf total carbon, **(I)** Leaf C:N ratio. *Means P<0.05, means P<0.01, * means P<0.001, ns means Not Significant.

NaCl treatment and inoculation with strains P6, N20, and N21 had significant effects on C, N content, and C: N ratio in various organs of *R. soongorica* seedlings (see **Figure 4**). The C contents in leaves and stems of *R. soongorica* seedlings were higher than that in roots, and the N contents in roots were higher than that in stems and leaves. Inoculated with strains P6, N20, and N21, the N contents in roots and leaves, and the C contents in leaves of *R. soongorica* seedlings increased. Among them, the N contents in roots of seedlings were significantly increased by 20.59%, 14.19%, and 8.33% (*P<*0.05), and the C contents in leaves increased by 2.78%, 3.41%, and 2.55% (*P<*0.05), compared with the control (CK). Additionally, the C: N ratio in the roots of seedlings decreased by 16.64%, 12.74%, and 7.41% (*P<*0.05). Under NaCl stress, the N in roots stems and leaves of *R. soongorica* seedlings increased by 59.52%, 25.07%, and 22.04%, and the C in the roots, stems and leaves decreased by 10.46%, 3.12%, and 3.88%, respectively, compared with the control (CK). Additionally, the C: N ratio of root, stem, and leaf decreased by 43.85%, 22.51%, and 21.24% respectively (*P<*0.05). However, after inoculation with P6, N20, and N21 strains, these change trends of the C, N contents, and C: N ratio in roots, stems, and leaves of *R. soongorica* seedlings under NaCl stress were reversed. When inoculated with P6, N20 and N21 strains, compared with NaCl treatment (S), the N contents in roots, stems, and leaves of *R. soongorica* under NaCl stress significantly decreased (*P<*0.05), the C contents in roots, stems, and leaves of seedlings significantly increased (*P<*0.05), the C: N ratio in roots, stems, and leaves of seedlings significantly increased(*P<*0.05). The effects of NaCl stress on the contents of the C, N, and the ratio of C: N in the roots, stems and leaves of *R. soongorica* seedlings were very significant, inoculated with *Enterobacter* P6, N20, and N21 could weaken the effects of salt stress. These Indicate that *Enterobacter* P6, N20, and N21 can promote plant growth and alleviate salt stress by regulating the distribution of nutrients distribution of *R. soongorica* seedlings.

### 3.6 Relationships between biomass and the C, N contents of R. soongorica seedlings

**Figure 5:**
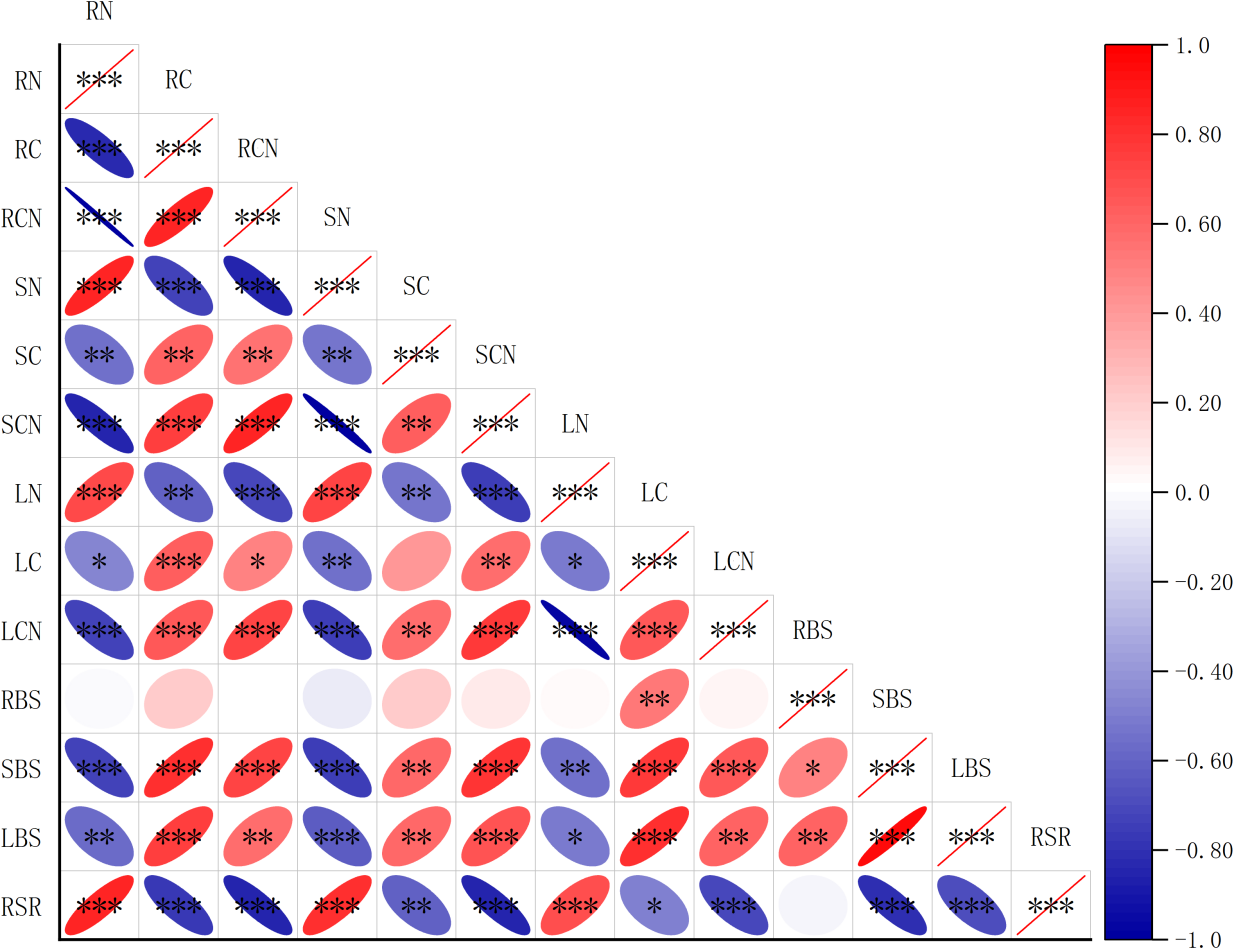
Spearman analysis between biomass and the C, N contents, and C: N ratio in different organs of *R. soongorica*. RN is the N content of root; RC is the C content of root; RCN is the C: N ratio of the root; SN is the N content of stem; SC is the C content of stem; SCN is the C: N ratio of stem; LN is the N content of leaf; LC is the C content of leaf; LCN is the C: N ratio of leaf; RBS is root biomass; SBS is stem biomass; LBS is leaf biomass; RSR is root-to-shoot ratio *Means P<0.05, ** means P<0.01, *** means P<0.001.

Correlation analysis showed that there is a strong association between biomass and the C, N contents, and C:N ratio in different organs of *R. soongorica* (**Figure 5**). The stem biomass was significantly positively correlated with the C contents of roots, stems, and leaves (r= 0.87; r= 0.70; r= 0.82), and negatively correlated with the N contents of roots, stems, and leaves (r= -0.81; r= -0.82; r= -0.70). The leaf biomass was significantly positively correlated with the C contents of rhizome, leaf, and stem (r= 0.79; r= 0.74; r= 0.85), and negatively correlated with the N contents of rhizome, leaf, and stem (r= -0.73); r= -0.74;r = -0.67). The C: N ratio of roots, stems, and leaves was significantly positively correlated with C contents of roots, stems, and leaves (r= 0.93; r= 0.71; r= 0.80), and significantly negatively correlated with N contents of roots, stems, and leaves (r= -0.99; r = -0.99;r =- 0.99). The root-to-shoot ratio was positively correlated with the N contents of roots, stems, and leaves (r= 0.88; r= 0.84; r= 0.69), and negatively correlated with the C contents of roots, stems, and leaves (r=-0.87; r =-0.62;r =-0.53). In conclusion, there is a close relationship between the biomass of roots, stems, and leaves and their C, N content and C: N ratio.

## 4 Discussion

### 4.1 *Enterobacter* plays a significant role in promoting healthy plant growth

*Enterobacter* is widely recognized for its potential to improve the soil environment and promote crop growth (Wang et al., 2022a). It is one of the widely studied PGPRs, which can increase the availability of soil nutrients (Roslan et al., 2022), regulate soil enzyme activities (Ji et al., 2024), facilitate biofilm formation (Li et al., 2022a), bolster resilience (Chen et al., 2024; Ji et al., 2020), and exert biological control (Wang et al., 2024) to promote the good growth of plants under stress. In this study, the P6 and N20 strains were identified as *E. hormaechei*, and the N21 strain was identified as *E. asburiae*. They can not only produce IAA but also solve phosphorus and fix nitrogen, which is the characteristic of ideal PGPR(see **Table 1**). Especially in the barren desert environment, they play a key role in promoting the growth of desert plants and enhancing their stress resistance. Studies have demonstrated that *Enterobacter cloacae* effectively enhances the growth of cucumber seedlings (Fan et al., 2024), cotton (Yue et al., 2023), and wheat (Ji et al., 2020), when grown under saline-alkali conditions. *E. hormaechei* can improve plant physiology and yield through soil phosphate (P) and potassium (K) amelioration (Ranawat et al., 2021a, 2021b; Roslan et al., 2022; Shahzad et al., 2020). *E. hormaechei* DS02Eh01 has the ability to promote plant growth and biofilm formation and has the potential for environmental ecological restoration (Li et al., 2022a). *E. asburiae* enhances salt tolerance and increases yield in rice (Ning et al., 2024), *Chenopodium quinoa* (Mahdi et al., 2020), and *Medicago sativa* (Li et al., 2024). It can be seen that *Enterobacter* plays an important role in promoting the healthy growth of plants. We also obtained similar results through pot experiments. Under NaCl stress, the height, basal diameter, and root surface area of *R. soongorica* seedlings were significantly reduced compared with the control (CK). However, inoculation with strains P6, N20, and N21, the seedling height, basal diameter, and root surface area of *R. soongorica* seedlings under NaCl stress were significantly higher than those of NaCl-treated seedlings. The results showed that inoculation with *Enterobacter* could promote the growth and enhance salt tolerance of *R. soongorica* seedlings.

### 4.2 *Enterobacter* promote plant growth and enhance salt tolerance by regulating biomass allocation

Plant organs serve distinct biological functions: leaves fix new carbohydrates, roots absorb water and nutrients and stems offer support and transportation pathways. The rational allocation of biomass among these organs is crucial in driving resource acquisition and facilitating growth (Poorter et al., 2011). At present, many studies on biomass allocation have been widely reported. Biomass allocation is affected not only by plant species and individual development but also by environmental conditions (Rutishauser et al., 2019). The study revealed that under low light and CO2 conditions, the proportion of biomass allocated to aboveground parts of plants was higher than that of roots (Guo et al., 2016). When plants are exposed to water and nutrient stress, more biomass is allocated to the plant root system (Grechi et al., 2007). This study found that under NaCl stress, the biomass of roots, stems and leaves of *R. soongorica* seedlings was significantly reduced compared with the control (CK), but the root-to-shoot ratio was significantly increased by 99.20%. Salt stress negatively affects the biomass in roots, stems, and leaves of *R. soongorica*. However, *R. soongorica* seedlings allocate more resources to roots, increase the proportion of root biomass, and increase the root surface area to enhance nutrient absorption. Under drought conditions, more biomass will be allocated to the roots of five tropical tree species, which is consistent with our research results (Asefa et al., 2022). This shows that plants respond to environmental changes by adjusting the allocation of biomass to different organs, prioritize the allocation of biomass to organs that have access to more limiting resources in order to promote growth (Guo et al., 2016), which is consistent with the optimal partitioning theory.

Microbial inoculants can resist biotic and abiotic stresses by regulating the biomass allocation. Inoculation of *Enterobacter cloacae* QZS3 significantly regulated *Sorghum bicolor* biomass and promoted plant growth under cadmium stress (Chen et al., 2024). PGPR and AMF inoculation can largely or at least partially offset the detrimental effects of drought on biomass production, water relations, and nutrient dynamics of *Myrtus communis* seedlings (Azizi et al., 2022). In our study, inoculation with strains P6, N20, and N21 can increase the biomass of roots, stems, and leaves of *R. soongorica* seedlings, both with and without NaCl stress. Notably, the greatest biomass increase was observed in the roots, while the smallest increase was seen in the stems. Similar results were reported that the dry weight significantly increased in the shoot and root of mustard under saline conditions after inoculation of salt-tolerant *Pseudomonas* JMM15 and *Pseudomonas aeruginosa* HMM57 strains (Phour and Sindhu, 2020). PGPR can stimulate root development, form lateral roots and adventitious roots, and promote plant growth by producing exogenous IAA and other plant growth hormones (Li et al., 2022b). We selected three strains of *Enterobacter*, all produced IAA, which may be one of the reasons why the root biomass and root surface area of *R. soongorica* seedlings increased significantly compared with the control. Inoculated with strains P6, N20, and N21, the root-to-shoot ratio of *R. soongorica* seedlings significantly increased. This indicates that the proportion of root biomass allocation increased, ensuring that plants can obtain more nutrients under salt stress. Our research results were consistent with those of Ma and Wang (2020) they found that the root growth rate was faster than that of aboveground in desert plant *Salsola passerina*. The results indicated that *R. soongorica* seedlings responded to salt stress by adapting their biomass allocation as an adaptive strategy. Inoculation with *Enterobacter* could increase the biomass of roots, stems, and leaves of *R. soongorica* seedlings, adjust the biomass allocation to promote growth and enhance salt stress tolerance.

### 4.3 *Enterobacter* promote plant growth and enhance salt tolerance through nutrient partitioning

Carbon (C) and nitrogen (N) are key elements for plant growth and development (Chen and Chen, 2021). C provides the structural basis of plants and is the largest component of plant biomass, accounting for 50% of plant dry weight (Ågren, 2008). N is a structural component of amino acids and a component of all enzymes and is involved in many physiological processes (Seepaul et al., 2019). C and N assimilation are two of the most important physiological processes associated with plant growth and productivity (Otori et al., 2017). A pot experiment was conducted to study the effects of NaCl stress and PGPR inoculums on C and N contents in different organs of *R. soongorica* seedlings. The results showed that under both salt stress and normal conditions, the N contents in roots and leaves of *R. soongorica* seedlings were higher than that in stems, and the C contents in leaves and stems of *R. soongorica* seedlings were higher than that in roots. Plant organs collaborate to accomplish various physiological and biochemical reactions as an integrated system (Zhang et al., 2018). The root and leaf exhibit a source-sink relationship, where the leaf serves as the source of C. The fixed C in the leaf is preferentially allocated to the organ closest to the nutrients, prior to being transported to other organs (Li et al., 2022c). Salt stress usually inhibits nitrogen and carbon metabolism in plants. The significant decrease of C content in the roots, stems and leaves of *R. soongorica* seedlings may be attributed to salt stress inhibiting plant photosynthesis and reducing carbon fixation (Rahnama et al., 2010). In addition, plants must expend extra energy to overcome salt stress (Chaves et al., 2009). However, the N contents were significantly higher than that of the control in roots, stems, and leaves of *R. soongorica* under salt stress. One reason is the accumulation of non-protein nitrogen in saline plants under salt stress (Pessarakli et al., 2012), that these non-protein forms of nitrogen play a crucial role in osmoprotection and osmoregulation (Xie et al., 2022). Another reason may be that the increase in enzyme amount per leaf area leads to increased N content of *R. soongorica* under salt stress, but, the increase of enzymes involved in photosynthesis is not enough to compensate for the decrease in enzyme activity (Wang et al., 2022b). Wang et al. (2015) observed 18 desert halophytes and found that the leaf N content of desert halophytes was higher than that of desert grassland plants, this is consistent with our results. Therefore, increasing leaf nitrogen content should be a common strategy for desert saline plants.

Inoculation of rhizosphere microorganisms can change the contents of C and N in plants, help overcome biotic and abiotic stress, as well as improve growth and nutrition in plants (Mariotte et al., 2017). For example, AMF plays a key role in nutrient uptake in the soil-plant continuum, increasing plant tolerance to drought (Xiao et al., 2023). Inoculated with PGPR, *Sulla carnosa* showed different C: N:P stoichiometry under salt stress, which indicated that PGPR could improve plant tolerance to salt stress through nutrient allocation mode (Hidri et al., 2019). Our study proved this point, inoculated with strains P6, N20, and N21 and the N content in the root of *R. soongorica* increased significantly compared with the control (CK), and the C content in the leaf increased significantly. When inoculated with P6, N20, and N21 strains, compared with NaCl treatment (S), the N contents in roots, stems, and leaves of *R. soongorica* under NaCl stress significantly decreased, the C contents in roots, stems, and leaves of seedlings significantly increased. Similar results were reported previously that inoculation AMF the N and P contents significantly increased in *Cinnamomum migao* seedlings, with the C contents in leaves and stems of plants higher than in control plants (Xiao et al., 2023). Therefore, it is a feasible adaptive strategy to use PGPR to maintain the nutrient balance of plants in desert areas (Elser et al., 2003).

Plant C: N ratios are important indicators for exploring elemental allocation and adaptation strategies in plants (Zhang et al., 2020). An excessively high or low C: N ratio can impede the absorption of nutrients by plants, whereas a suitable C: N ratio can enhance the absorption and utilization of nutrients by plants. Previous studies showed that plants generally have a higher C: N ratio under drought stress (Jiang et al., 2021). Our study demonstrated that the C: N ratio of roots, stems and leaves of *R. soongorica* seedlings under salt stress was significantly lower than that of the control, but inoculated with strains P6, N20, and, N21, the C: N ratio of roots, stems and leaves significantly increased. C: N:P chemometrics of *Trifolium repens* inoculated with AMF have been studied, and consistent results have been observed (Dong et al., 2023). A higher C: N ratio indicated higher nitrogen use efficiency, which made the seedlings grow normally under salt stress.

### 4.4 Correlation of biomass and elemental allocation of *R. soongorica* seedlings

The biomass of roots stems, and leaves is closely related to the C and N content of R. soongorica seedlings. Understanding the correlation between the C and N element content in plant leaves and biomass is crucial (Song et al., 2024). Studies have shown that the N in leaf content can influence the final allocation of plant biomass(Ding et al., 2011; Xu and Jiang, 2015); The improvement of biomass in mycorrhizal plants may also be due to the enhanced N uptake(Habibzadeh et al., 2013). This study found that root biomass was significantly positively correlated with leaf C content, stem biomass was significantly positively correlated with root stem leaf stem C content, significantly negatively correlated with root stem leaf stem N content, leaf biomass was significantly positively correlated with root stem leaf C content, and significantly negatively correlated with root stem leaf N content. The results indicated a significant correlation between the content of C and N in the roots, stems, and leaves of R. soongorica seedlings and their biomass allocation. Investigating this relationship was crucial for comprehending plant nutrient utilization strategies and the elemental balance within the plant.

## 5 Conclusion

*R. soongorica*, a desert plant, was selected as the subject of research in this study. A pot experiment was performed to evaluate how three strains of Enterobacter could enhance the growth of *R. soongorica* seedlings when exposed to NaCl stress. The study investigated the impact of NaCl stress and *Enterobacter* inoculation on biomass allocation as well as the C and N content in the root, stem, and leaf of *R. soongorica* seedlings. The findings revealed that NaCl stress significantly reduced plant height, basal stem diameter, root surface area, biomass, carbon content, and C: N ratio *R. soongorica* seedlings compared to the control (CK). Conversely, the root-to-shoot ratio and the N content of these seedlings increased significantly (*P<*0.05). However, upon inoculation with strains P6, N20, and N21, there was a considerable improvement in plant height, basal diameter, root surface area, biomass, the carbon content of roots and leaves, as well as the C:N ratio, compared to the NaCl-treated group (S). In contrast, the nitrogen content of the seedlings decreased significantly (*P*<0.05). The results indicated that under salt stress, *R. soongorica* seedlings responded by adjusting biomass allocation among roots, stems, and leaves, as well as by modifying their carbon and nitrogen content. Inoculation with *Enterobacter* further augmented the biomass of these seedlings, particularly in the roots, ensuring that the plants could access more nutrients to mitigate the harmful effects of salt stress. This observation aligns with the principles of optimal allocation theory. Additionally, Enterobacter inoculation had a profound impact on the carbon and nitrogen content, as well as the C:N ratio, in the seedlings’ roots, stems, and leaves. This adjustment in nutrient distribution promoted the seedlings’ growth and enhanced their salt tolerance. Although a strong correlation was observed between the biomass and the carbon and nitrogen content of *R. soongorica* seedlings, further research is needed to explore the relationship between these factors and the carbon and nitrogen content of the soil.

## Data Availability

The datasets presented in this study can be found in online repositories. The names of the repository/repositories and accession number(s) can be found in the article/supplementary material.
